# Inhibition of cell growth and invasion by epidermal growth factor-targeted phagemid particles carrying siRNA against focal adhesion kinase in the presence of hydroxycamptothecin

**DOI:** 10.1186/1472-6750-8-74

**Published:** 2008-09-18

**Authors:** Xiu-Mei Cai, Hai-Long Xie, Ming-Zhu Liu, Xi-Liang Zha

**Affiliations:** 1 Department of Biochemistry and Molecular Biology, Shanghai Medical College, Fudan University, Shanghai, PR China; 2 Institute of Cancer Research, South China University, Hengyang, PR China

## Abstract

**Background:**

Previous studies demonstrated the EGF-targeted phagemid particles carrying siRNA against Akt could be expressed efficiently in the presence of hydroxycamptothecin (HCPT). However, no significant cell growth inhibition was obtained. This study was to further investigate whether the EGF-targeted phagemid particles carrying siRNA would be a promising tool for anti-cancer siRNA delivery.

**Results:**

We found that pSi4.1-siFAK phagemid particles could significantly inhibit the expression of focal adhesion kinase in the HCPT-treated cells. Moreover, we also observed that the particles could potently suppress cell growth and cell invasion.

**Conclusion:**

These results indicated that EGF-targeted phagemid particles might be a promising tool for anti-cancer siRNA delivery in the presence of HCPT.

## Background

Small interfering RNA (siRNA) molecules are capable of interrupting the translation of a specific protein by inducing post-transcriptional gene silencing. It is a promising method for silencing therapeutic target genes. A variety of delivery systems are proposed for the delivery of siRNA into cells *in vitro *and *in vivo*. Since phage-based vectors do not exhibit natural tropism towards mammalian cells and can be genetically modified for specific applications, modified phage-based vectors are an attractive alternative strategy for gene delivery. They have been successfully modified to deliver genes to target cells by the effective use of targeting ligands such as growth factors, antibodies, and viral capsid proteins [1-7]. To increase the density of ligand display on the phages, an epidermal growth factor (EGF)-modified helper phage genome M13EGFKO7CT was established, which could produce EGF-targeted phagemid particles [8]. The phagemid particles could deliver reporter genes into target cells; however, the efficiency of delivery was limited [8]. A topoisomerase I inhibitor such as camptothecin or hydroxycamptothecin (HCPT) could substantially enhance the transduction of the phagemid gene delivery particles [1,9]. The recent studies showed that the cell-targeted phagemid particles could efficiently deliver siRNA against Akt into cell in the presence of HCPT [10]. But, no significant growth inhibition was observed. Thus, to be an effective anti-cancer siRNA delivery vector, more studies should be performed, such as carrying siRNA against other oncogenes.

Focal adhesion kinase (FAK), a non-receptor tyrosine kinase, has been implicated in several cellular processes such as proliferation, apoptosis, motility, and invasion. Increased expression of FAK has been found in various malignant tumors, including tumors derived from the lungs, breasts, head and neck, and ovaries [11-14]. Therefore, FAK is recognized as an important therapeutic target in the treatment of cancer. Delivery of siFAK by lipofectamine could significantly block the expression of FAK and trigger cell death and block cell migration [15]. But, the siFAK could not be delivered to target cells. To further investigate whether the EGF-targeted phagemid particles in combination with RNA interference (RNAi) would represent an effective therapeutic approach, we used phagemid particles carrying siRNA against FAK to infect H1299 cells and examined the therapeutic potential of this approach.

## Results and Discussion

**Figure 1:**
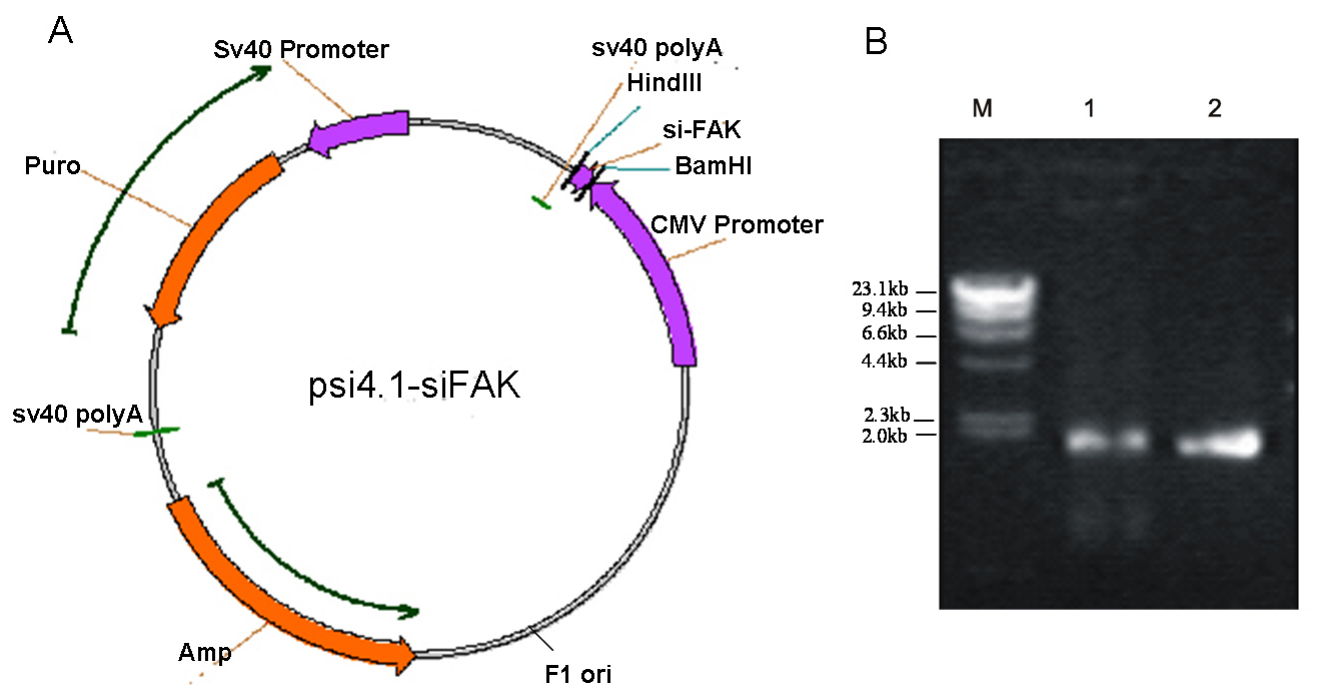
**The map of pSi4.1-siFAK phagemid particle and analysis of the ssDNA released from phagemid particles**. A: pSi4.1-siFAK phagemid particle was constructed as follows: the shRNA against FAK was inserted into the modified pSilencer4.1 vector digested with BamHI and HindIII. B: ssDNA was purified from phagemid particles to analyze the ratio of phagemids to helper phage genomes packaged in the phagemid particles. The results indicated that almost all the DNA packaged comprised phagemids. Lane M: λ DNA/HindIII; Lane 1: pSi4.1-siFAK phagemid particles; Lane 2: pSi4.1-simock phagemid particles.

**Figure 2:**
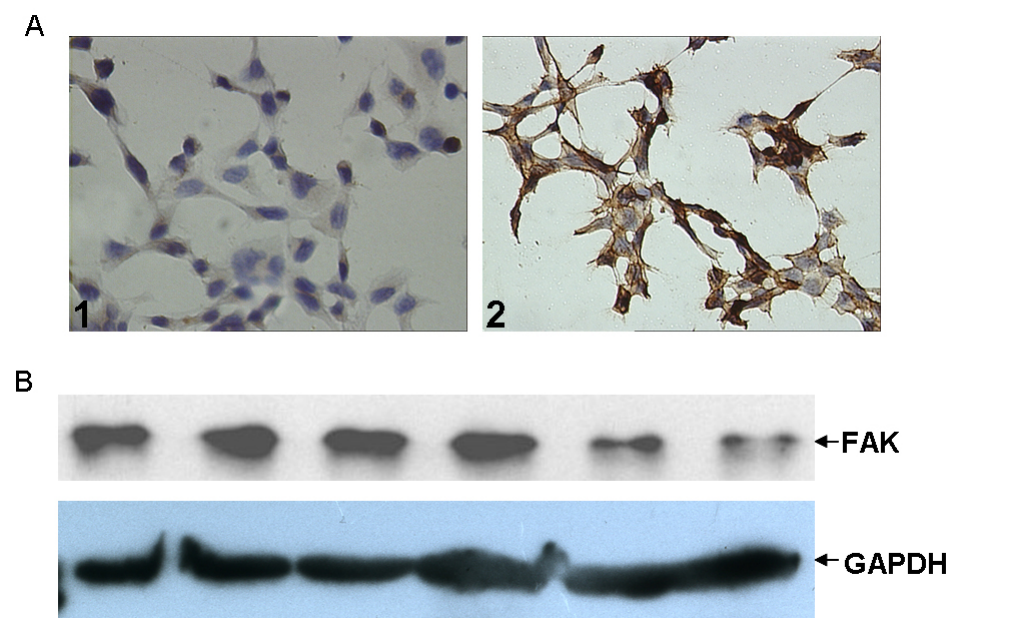
**Immunocytochemistry assay of EGFR expression and Western-blot analysis of the specific FAK gene silencing by EGF-targeted phagemid particles mediated RNA interference**. A: In the immunocytochemical assay, H1299 cells showed a strong positive EGFR immunoreactivity, while very light immunostaining was observed in the U87 cells that were used as negative controls. 1, H1299; 2, U87. B: H1299 cells were infected with pSi4.1-siFAK phagemid particles. In HCPT treated-cells, the pSi4.1-siFAK phagemid particles could inhibit FAK expression to a great extent. 1, H1299; 2, H1299 infected with pSi4.1-simock phagemid particles; 3, H1299 infected with pSi4.1-simock phagemid particles in the presence of 2.5 μM HCPT; 4, H1299 infected with pSi4.1-siFAK phagemid particles; 5, H1299 infected with pSi4.1-siFAK phagemid particles in the presence of 2.5 μM HCPT; 6, H1299 transfected with pSi4.1-siFAK using Lipofectamine 2000.

Previous studies showed that the cell-targeted phagemid particles were efficient siRNA delivery vectors in the presence of HCPT and they could efficiently deliver siRNA against Akt into targeted cells in the presence of HCPT [10]. But, no significant growth inhibition was observed. Thus, to be an effective anti-cancer siRNA delivery vector, more studies should be performed, such as carrying siRNA against other oncogenes. In this study, we made phagemid particles carrying siRNA against FAK to infect H1299 cells and examined the therapeutic potential of this approach. First, the short hairpin RNA (shRNA) against FAK was subcloned into pSi4.1CMV-f1, thus forming pSilencer4.1-siFAK (pSi4.1-siFAK) (Fig. 1A). Then, we purified ssDNA from phagemid particles to analyze the ratio of phagemids to helper phage genomes packaged in the phagemid particles. The results indicated that almost all the DNA packaged comprised phagemids (Fig. 1B). Previously, the modified helper phage genome (plasmid) M13EGFKO7CT was created to produce EGF-targeted phagemid particles [8,10]. The M13EGFKO7CT plasmid was used to package pSi4.1-siFAK phagemid particles, following which the phagemid particles displayed the EGF ligand. In the immunocytochemical assay, we found that H1299 cells showed a strong positive EGFR immunoreactivity, while very light immunostaining was observed in the U87 cells that were used as negative controls (Fig. 2A). Therefore, we infected H1299 cells with pSi4.1-siFAK phagemid particles. Western blotting assay showed that the pSi4.1-siFAK plasmid transfected by lipofectamine could significantly block the expression of FAK. This was not observed in cells transduced with pSi4.1-siFAK phagemid particles without HCPT treatment. Surprisingly, in HCPT treated-cells, the pSi4.1-siFAK phagemid particles could inhibit FAK expression to a great extent. Inhibition of FAK expression was not found in the cells infected with mock phagemid particles (Fig. 2B). Taken together, the vectors could deliver siRNA to human carcinoma cells efficiently in the presence of HCPT. HCPT had been shown to increase the efficiency of transduction of the phagemid vectors [1,9,16]. However, the mechanism by which HCPT increased transgene expression was not fully understood [1,9]. It was thought to involve the activation of the host cell repair machinery in response to DNA damage [1,16,17]; however, further studies are required to confirm this.

**Figure 3:**
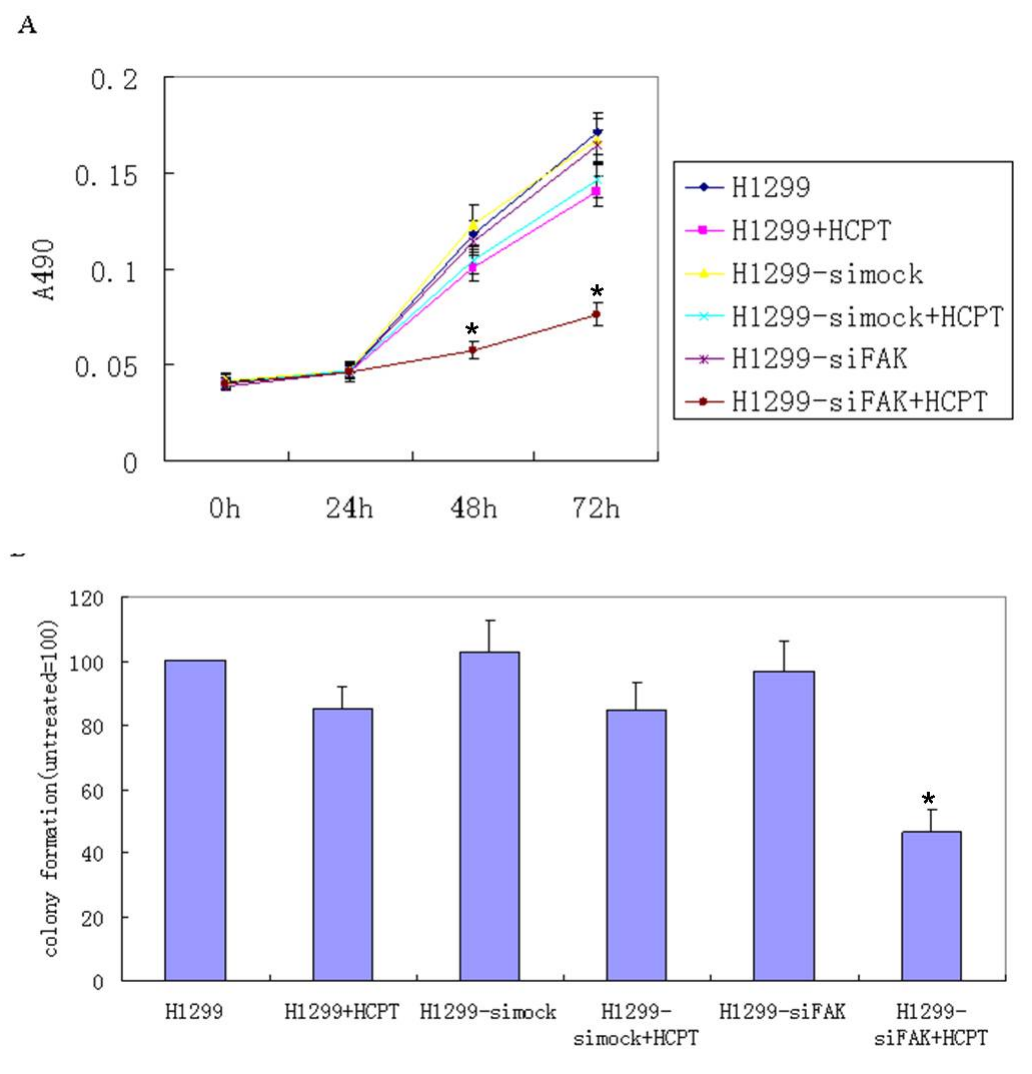
**Phagemid particles of pSi4.1-siFAK could inhibit H1299 cell growth**. A: MTT assay. B: Colony-Forming Ability Assay. The results shown were representative of at least three independent experiments. The HCPT-treated cells that were infected with pSi4.1-siFAK phagemid particles exhibited a significant inhibition of proliferation (*, p < 0.01) compared with H1299 cells.

A series of experiments were performed to determine whether the pSi4.1-siFAK phagemid particles could arrest H1299 cell growth. Cell growth was monitored by the MTT assay, and it was observed that all the cells grew at a similar rate at 0 and 24 h. However, a great difference began to appear at 48 and 72 h. In the presence of HCPT, the inhibitory rates were approximately 12–19% in parent and mock cells, compared with parent cells without HCPT. Amazingly, the inhibitory rates reached approximately 52–61% in HCPT-treated cells infected with pSi4.1-siFAK phagemid particles, compared with the control cells. No significant growth inhibition was found in the cells infected with the mock vector or pSi4.1-siFAK phagemid particles in the absence of HCPT (Fig. 3A). In addition, we examined the growth arrest of the pSi4.1-siFAK phagemid particles at the 3-dimensional level. In the colony-forming ability assay, the number of colonies of pSi4.1-siFAK phagemid particles decreased by almost 54% in the presence of HCPT, compared with the control cells. In contrast, cells in other groups exhibited little change in the number of colonies (Fig. 3B). These data suggested that the treatment of pSi4.1-siFAK phagemid particles could dramatically reduce cell viability in the presence of HCPT.

**Figure 4:**
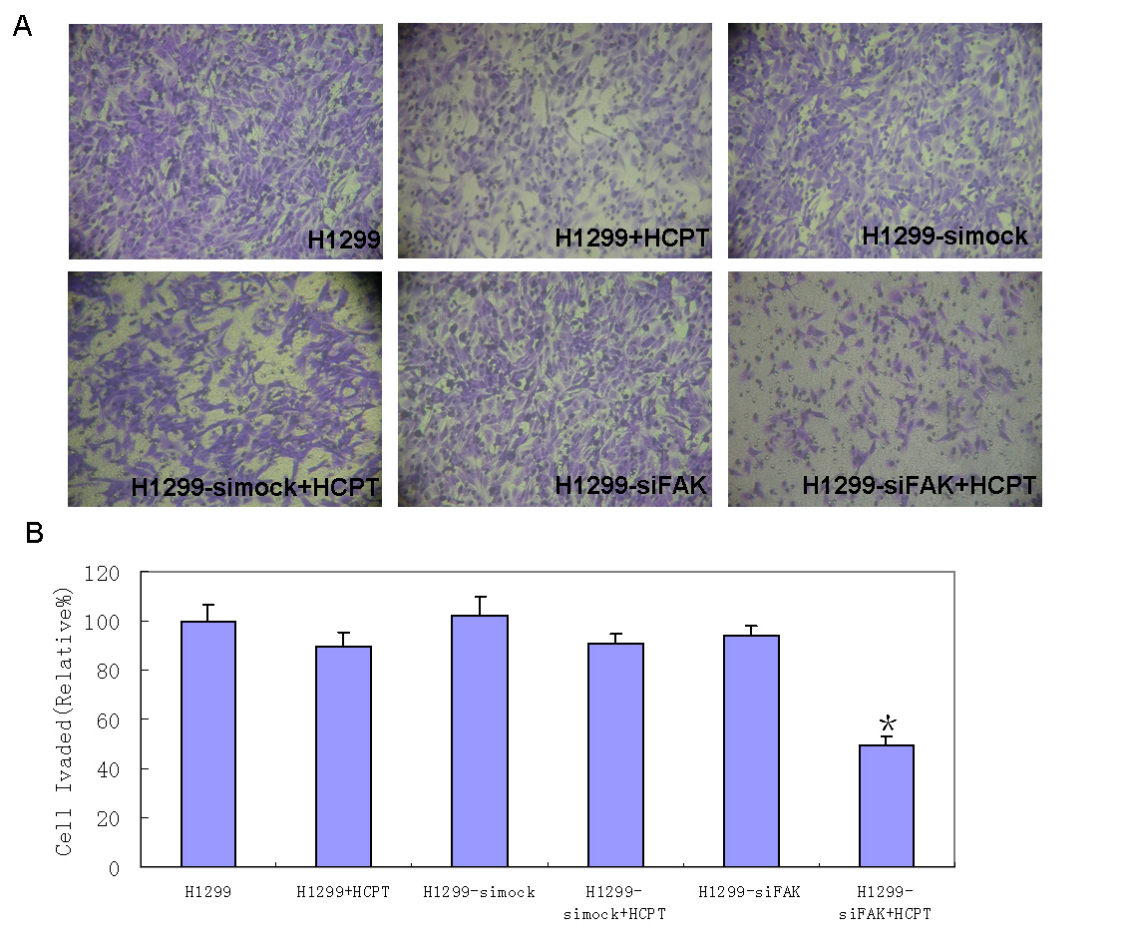
**Phagemid particles of pSi4.1-siFAK could inhibit H1299 cell invasion**. A: Cells that invaded after 36 hr through ECM-Gel-coated transwell inserts were stained with crystal violet stain. B: Cell invasion assay. The HCPT-treated cells that were infected with pSi4.1-siFAK phagemid particles exhibited a significant inhibition of invasion (*, p < 0.01) compared with H1299 cells.

Furthermore, we quantified the effect of pSi4.1-siFAK phagemid particles on cell invasion. In the transwell invasion assay, the number of pSi4.1-siFAK phagemid particles transduced-cells (HCPT treatment) invading through the membrane coated with ECM gel was less than that of the control cells (Fig. 4A). The cell invasion was markedly reduced by approximately 50% in pSi4.1-siFAK phagemid particles transduced-cells (HCPT treatment), compared with the control cells; the other groups however showed no obvious change (Fig. 4B). Thus, the above data indicated that the transfection of H1299 cells with pSi4.1-siFAK phagemid particles and the HCPT treatment could dramatically inhibit cell invasion.

RNAi has revolutionized the biological sciences because it can selectively silence messenger RNA (mRNA) expression. However, the delivery of this RNA into target cells represents the main barrier for using siRNA as a novel drug against tumor targets. Since filamentous phages only showed tropism for cells that expressed the appropriate receptors, this tropism could be conferred to phage particles by the expression of a targeting ligand on the phage coat [3,4,6]. Therefore, phages would be an attractive alternative strategy for siRNA delivery. This study demonstrated that EGF-targeted phagemid particles in combination with RNAi and the HCPT treatment represent a new therapeutic approach for silencing oncogenes. However, in order to act as a cancer gene-delivery vector, phage vectors should display other targeting ligands to increase their specificity for different types of tumors.

## Conclusion

Therefore, phages would be an attractive alternative strategy for siRNA delivery. This study demonstrated that EGF-targeted phagemid particles in combination with RNAi and the HCPT treatment represent a new therapeutic approach for silencing oncogenes. However, in order to act as a cancer gene-delivery vector, phage vectors should display other targeting ligands to increase their specificity for different types of tumors.

## Methods

### Reagents

Dulbecco's Modified Eagle's Medium (DMEM), Dulbecco's phosphate-buffered saline and fetal bovine serum (FBS) were obtained from Invitrogen (Grand Island, USA). Restriction endonucleases were obtained from TaKaRa Biotechnology (Dalian, China).

### Cell culture

H1299 (human lung carcinomas) cells and U87 (human glioblastoma) cells were cultured at 37°C in DMEM containing 10% FBS in a humidified atmosphere containing 5% CO2.

### Plasmid construction

The modified pSilencer4.1 plasmid was obtained from Dr. Z. Li (Shanghai Jiao Tong University, Shanghai, China). FAK siRNA (target sequence, 5'-GAACCTCGCAGTCATTTAT-3') has been proven to be effective for inhibiting FAK [18]. A 59-nt oligo-DNA duplex (5'-AGCTTGAACCTCGCAGTCATTTATTTCAAGAGAATAAATGACTGCGAG GTTCTTTTTTG-3'/5'-GATCCAAAAAAGAACCTCGCAGTCATTTATTCTCTTG AAATAAATGACTGCGAGGTTCA-3') was inserted into the pSilencer4.1 vector digested with BamHI and HindIII. The mock siRNA sequence is 5'-GTCTCCGAACGTGTCACGT-3' [18]. Another 59-nt oligo-DNA duplex (5'-AGCTTGTCTCCGAACGTGTCACGTTTCAAGAGAACGTGACACGTTCGGAGACTTTTTTG-3'/5'-GATCCAAAAAAGTCTCCGAACGTGTCACGTTCTCTTGAAACGTGACACGTTCGGAGACA-3') was inserted into the modified pSilencer4.1 vector digested with BamHI and HindIII.

### Preparation of phagemid particles

Briefly, M13KO7EGFCT was transformed into *Escherichia coli *to create LMP cells [8]. The phagemid carrying siFAK was transformed into the LMP cells. The cells were then plated on Luria-Bertani (LB) agar containing 70 μg/mL kanamycin and 50 μg/mL ampicillin and incubated at 37°C overnight. A cell clone was picked up and transferred into 1 L of LB solution containing 70 μg/mL kanamycin and 50 μg/mL ampicillin. After shaking at 37°C for 15 h, the supernatant of the culture was collected and the phagemid particles were purified with polyethylene glycol (PEG)/NaCl precipitation. Then, they were quantified by ELISA as previously described [19].

### Purification of single-stranded DNA from phagemid particles

Single-stranded DNA (ssDNA) was extracted from the phagemid particles by using the Ph.D.-12 phage display peptide library kit (New England Biolabs, USA) according to the manufacturer's protocol. Briefly, the phagemid particles were precipitated by PEG/NaCl. The pellet was then suspended in iodide buffer. Ethanol (250 ml in total) was added to the buffer, and the pellet was incubated in it for 10 min. The pellet was collected after centrifugation at 12 000 × *g *for 10 min. It was finally dissolved in 30 μL Tris-EDTA (TE) buffer (10 mM Tris-HCl [pH 8.0] and 1 mM EDTA) and analyzed by agarose gel electrophoresis.

### Immunocytochemistry

Monolayer cells were grown on glass cover slips and fixed with 4% paraformaldehyde. Endogenous peroxidase activity was quenched with 2% hydrogen peroxide in methanol for 45 min. After the cells were blocked with 5% normal serum for 30 min, they were incubated with primary antibodies to the epidermal growth factor receptor (EGFR) (Cell Signaling Technology, MA) diluted to 1:500 in phosphate-buffered saline (PBS) for 1 h at room temperature. Then, the cells were rinsed and incubated with horseradish peroxidase (HRP)-conjugated secondary antibodies (Watson Biotech, China) diluted to 1:500 in PBS for 1.5 h at room temperature. The reaction was developed with diaminobenzidine (Dako, Japan), and then the slides were counterstained with Mayer's hematoxylin. After a final wash, the slides were mounted and the cells were examined using an Olympus photomicroscope (400× magnification).

### *In vitro *phagemid particle transfection

The cells were plated onto 24-well plates at a density of 10 000 cells per well 24 h prior to the addition of phage particles. The phages were added at 10^11 ^pfu/mL and incubated with the cells for 48 h at 37°C in complete media. Then, the medium was removed and the cells were incubated in fresh medium containing 2.5 μM HCPT for 6 h at 37°C. Following this, the medium was replaced with fresh medium, and the cells were incubated for 18 h at 37°C. All the transfections were performed in triplicate at least 2 times.

### Western blot analysis

In total, 10^6 ^H1299 cells transfected with a variety of phagemid particles were lysed after the HCPT treatment. The cells were washed with PBS and lysed in a buffer containing 50 mM Tris (pH 7.5), 5 mM EDTA, 300 mM NaCl, 0.1% Igepal, 0.5 mM NaF, 0.5 mM Na_3_VO_4_, 0.5 mM phenylmethylsulfonyl fluoride, and an antiprotease mixture. Equal amounts of protein were loaded on a SDS-PAGE and transferred onto a nitrocellulose membrane. They were incubated with specific primary antibodies and then with HRP-conjugated secondary antibodies. Proteins were visualized by fluorography using an enhanced chemiluminescence system (Pierce Biotechnologies, USA). The anti-FAK antibody (Santa Cruz, CA) was used in 1:1000 dilutions. The monoclonal antibody to GAPDH was purchased from Kang-Chen Biotech (Shanghai, China). The secondary antibody conjugated with HRP was purchased from Watson Biotech (Shanghai, China).

### MTT assay

After the HCPT treatment, H1299 cells transfected with a variety of phagemid particles were seeded onto a 96-well plate overnight in DMEM containing 10% FBS, and then grown for 0, 24, 48, and 72 h. Methyl thiazolyl tetrazolium (MTT) (20 μL) solution (5 mg/mL in PBS) was added to each well and incubated for 5 h at 37°C. The solution was removed and 200 μL of dimethylsulfoxide (DMSO) was added to each well. These plates were vibrated gently for 10 min; they then underwent detection in the universal microplate reader at 490 nm.

### Colony-forming ability assay

The efficiency of colony formation was assayed in 35-mm dishes prepared with a lower layer of 0.8% agar (GIBCO/BRL) overlaid with 0.3% agar containing 2 × 10^4 ^suspended cells. After 5 days, growth was estimated under a Nikon inverted phase-contrast microscope, and individual colonies of more than 50 cells were counted [20].

### Transwell invasion assay

Polymerized gels were prepared by neutralization of extracellular matrix (ECM) gel (Sigma, USA) with cold DMEM. Cells in DMEM with 0.5% bovine serum albumin (BSA) were plated on the gel, and DMEM with 0.5% BSA and 0.5% FBS was added to the bottom of the chambers. Photographs were taken 36 h later to capture the cells that had invaded below the gel surface. The number of invading cells in 5 fields was counted under a 200× magnification. Each value represents the average of 3 individual experiments, and the error bars represent SD. p-values were calculated by the ANOVA test in SAS8.2 [21].

## Authors' contributions

XMC carried out the molecular genetic studies, participated in the preparation of phagemid particles and drafted the manuscript. HLX carried out the immunoassays and performed the statistical analysis. MZL participated in transwell invasion assay. XLZ conceived of the study, and participated in its design and coordination and helped to draft the manuscript. All authors read and approved the final manuscript.
